# Supplementary material for: A Traceable DNA-Replicon Derived Vector to Speed Up Gene Editing in Potato: Interrupting Genes Related to Undesirable Postharvest Tuber Traits as an Example
Source: Plants (Basel). 2021 Sep 10;10(9):1882. doi: 10.3390/plants10091882 (PMC8468489; doi:10.3390/plants10091882)
Supplement: Supplementary file 1 [file plants-10-01882-s001.zip › plants-1354105-supplementary.pdf]

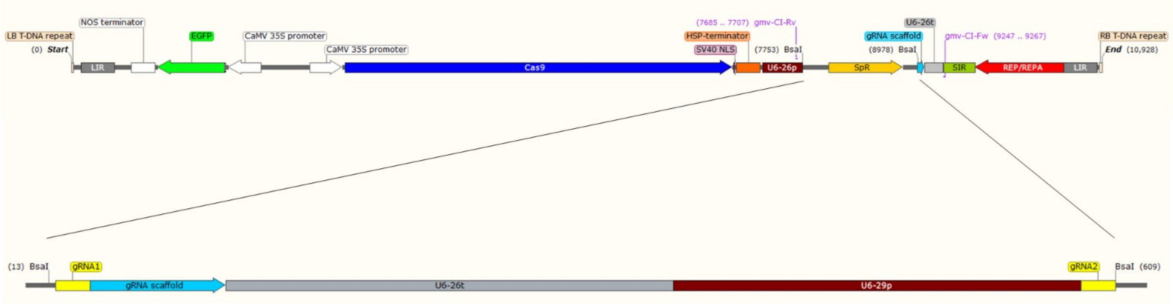

Figure S1. Assembly of the gRNA1-gRNA2 modules. Each selected gRNA pair (gRNA1 + gRNA2 for each gene) was amplified by PCR using primers according to Xing *et al.* [45] and cloned into pGEF-U by the Golden Gate reaction through cloning modules using *Bsa*I restriction sites to generate gene-specific pGEF-X editors (in which "X" represents either *POLYPHENOL OXYDASE* (*PPO*) *StPPO1*, *StPPO2* target genes, or Cold-induced Sweetening (CIS)-related *StvacINV1*, *StBAM1* target genes). Correct assemble was confirmed by sequencing using the gmv-CI-Fw/gmv-CI-Rv sequencing primers to confirm the two-guided module insertion into pGEF-U. The checked editing vectors were used in *Agrobacterium*-mediated gene transfer experiments of leaf explants of 'Yagana-INIA'. T-DNA RB, right border of the *Agrobacterium* T-DNA; LIR, large intergenic region from the *Bean yellow dwarf virus* (BeYDV); CaMV 35S, 35S promoter from *Cauliflower mosaic virus*; Cas9, Cas9 *Arabidopsis thaliana* codon usage; HSP terminator, terminator for *HEAT SHOCK PROTEIN 18.2* gene from *A. thaliana*; U6-26p, *A. thaliana* U6-26 RNA polIII promoter; U6-29p, *A. thaliana* U6-29 RNA polIII promoter; SpR, spectinomycin resistance gene; gRNA scaffold, gRNA scaffold sequence; U6-26t, *A. thaliana* U6-26 RNA polIII terminator; SIR, short intergenic region from BeYDV; Rep/RepA, nucleotide sequence for the Rep/RepA replication genes; EGFP, *Green Flourescent Protein* gene; LB T-DNA, left border of the *Agrobacterium* T-DNA; *Bsa*I, restriction enzyme sites for additional gRNA expression cassettes. gRNA1 and gRNA2, guide RNA sequences for each gRNA included.

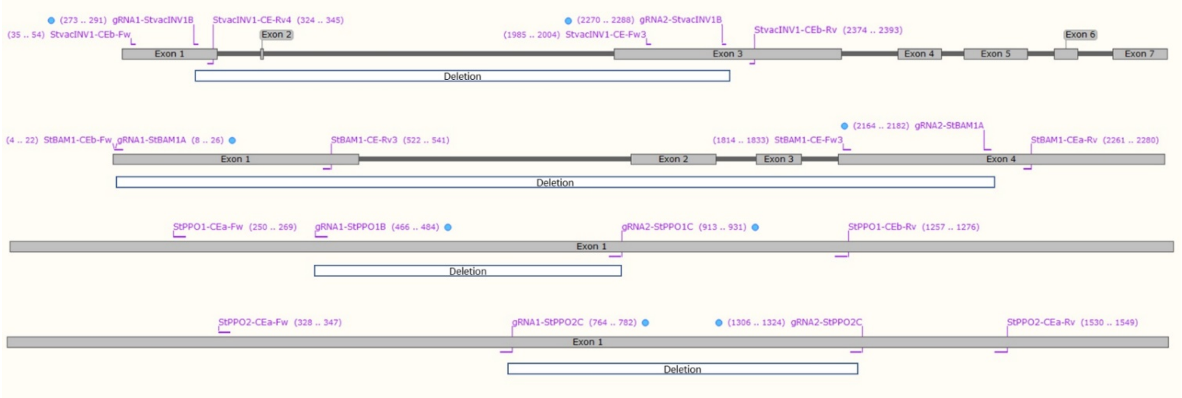

Figure S2. Target gene structures and expected deletions by double cut editing. Genes associated to the postharvest disorders were selected in this study: i.e., enzymatic browning associated to polyphenol oxidase (PPO) enzymes StPPO1 and StPPO2 and cold-induced sweetening process involving the vacuolar Invertase StvacINV1 and the Beta-amylase StBAM1 enzymes. The corresponding genomic sequences (Supplementary Table S2) and structures were established in the reference genome and processed by the "Potato CRISPR Search Tool". The target zones for gene editing (Deletion) were defined, leading the search tool to adjust the gRNA target zones upstream and downstream in a process running under "strict search" parameters. From this analysis, a list of gRNA pairs was obtained and the best gRNA pairs selected (blue dots) and assembled in particular versions in pGEF-U. Relevant primers used for characterizations are shown in purple.

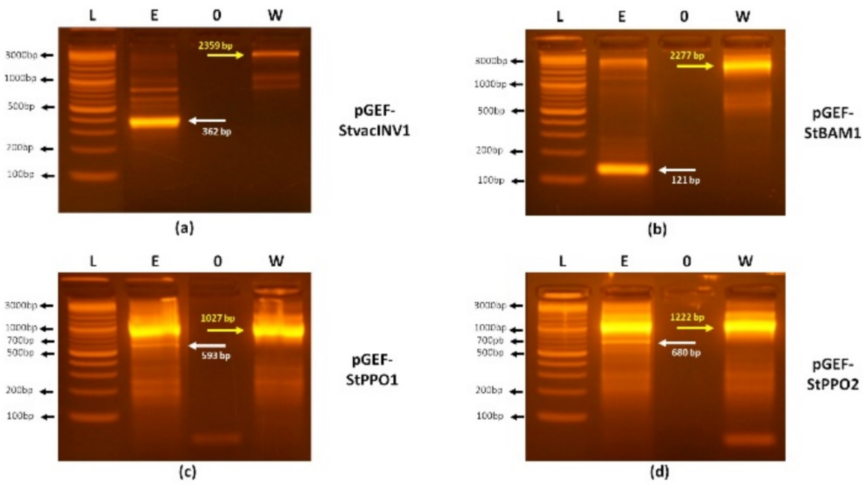

Figure S3. Targeted deletion editing capability of the gRNAs contained in each pGEF-X vector. Transient *Agrobacterium*-mediated gene transfer experiments of 'Yagana-INIA' leaf explants tracked 7 dpi and showing the highest GFP emission were subjected to genomic DNA isolations and PCR amplifications. Amplifications were carried out using the corresponding CE-primer pairs (Supplementary Table S6) and resolved by agarose gel electrophoresis and ethidium bromide staining. Double cut editing in the *StvacINV1* (**a**), *StBAM1* (**b**), *StPPO1* (**c**), and *StPPO2* (**d**) genes are shown. White arrows indicate smaller bands observed in explants subjected to pGEF-X editor treatment (E) compared to each original gene version observed wild type, non-edited explants (W, yellow arrows). Nuclease-free water used as PCR control (0).

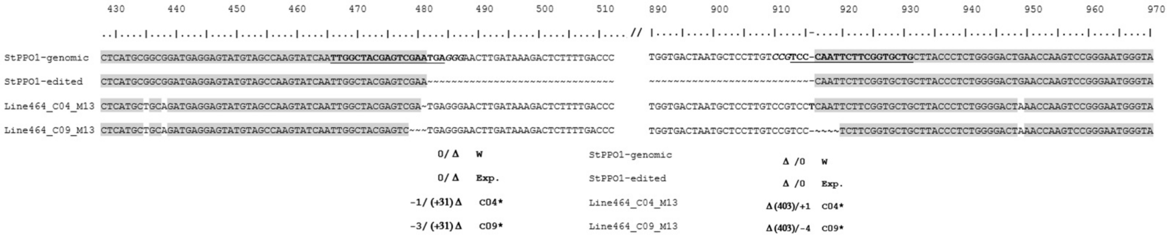

Figure S4. Analysis of the double cut editing in *StPPO1* gene in plant line #464. PCR characterization for double cut in the plant line #464 showed the expected size band (analyzed in Figure 5) plus other additional bands. Cloning and sequencing of the higher size bands in #464 showing wild type versions of the gene (genomic) compared with their theoretically edited (edited) and experimentally detected. Guide RNA sequences are in bold underlined fonts; protospacer adjacent motifs (PAM) are in bold italics fonts. Homologue nucleotides are boxed in grey. Nucleotide deletions are indicated by the "~" symbol; punctual nucleotide insertions are in bold fonts. Total insertions / deletions in the amplified fragments are indicated for each Line_x case. Δ, big deletions / insertions. Cx denotes the colony number of the sequenced bacterial clone resulting from the PCR band cloning process. *, representative result of two or more sequenced colonies.

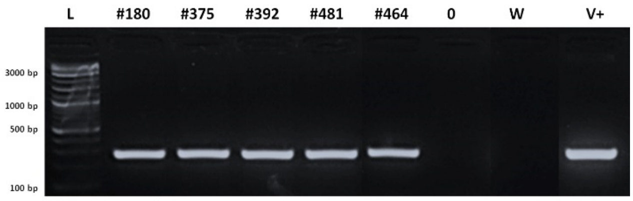

(a)

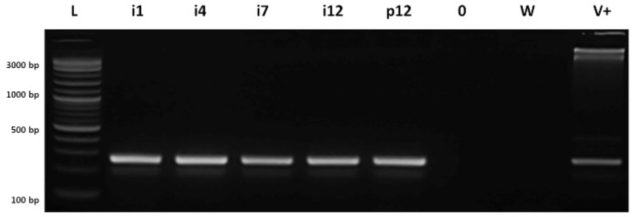

(b)

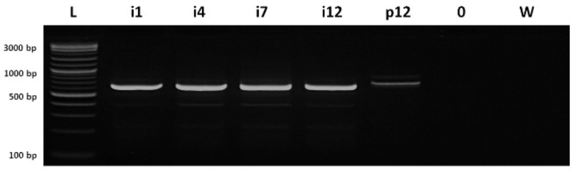

(c)

Figure S5. ***Green Fluorescent Protein*** **(*GFP*) gene insertion in the edited lines.** Total DNA isolations from the identified targeted deletion plant (**a**) and callus (**b**) lines were used in PCR amplifications assisted by the eGFP-nst1-fw and eGFP-nst1-rv primers, which amplify a 265 bp fragment. In addition, occurrence of recircularized geminivirus-replicon in the GFP emitting transgenic calli was evaluated by PCR amplification (**c**) using the pGEF-recircle-fw and pGEF-recircle-rv primers, which amplify a 626 bp fragment that includes the LIR element formed after rolling circle. Band identities were established by sequencing. #, number of the edited plant line; i, edited callus line using pGEF-StvacINV1); p, edited callus line using pGEF-StPPO2. 0, nuclease-free water; W, wild type 'Yagana-INIA' DNA; V+, pGEF-U vector. Primers in Supplementary Table S4.

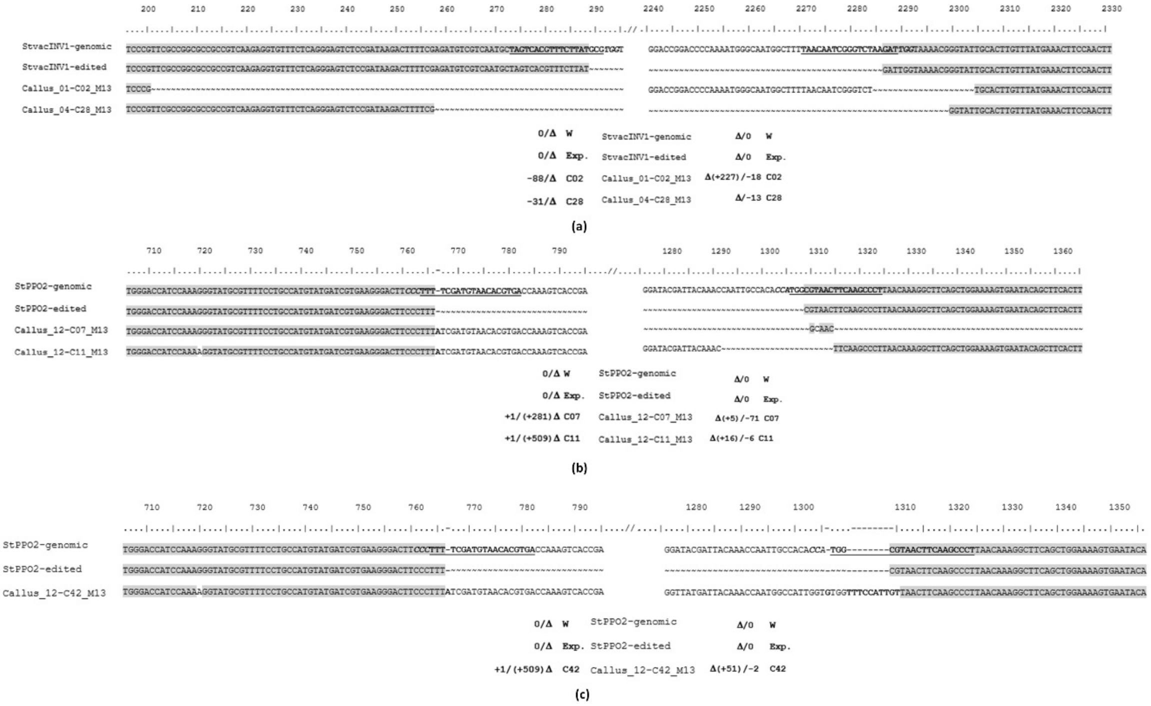

Figure S6. Gene editing characterization in calli lines stably expressing GFP. Calli lines obtained as the only product after more than 40 weeks of selection from experiments in the case of *StvacINV1* and *StPPO2* gene editing, were molecularly characterized for editing condition. Genomic DNA was extracted and subjected to PCR using the characterization primers. The amplification products were resolved by gel electrophoresis, and the smaller bands, in comparison to each original gene version, were cloned and sequenced. Different DNA repairs. Wild type versions of the genes (genomic) are compared with their theoretically edited (edited) and with experimentally detected versions in each GFP callus line (Line_x). Guide RNA sequences are in bold underlined fonts; protospacer adjacent motifs (PAM) are in bold italics fonts. Homologue nucleotides are boxed in grey. Nucleotide deletions are indicated by the "~" symbol; nucleotide insertions are in bold fonts. Total insertions / deletions in the amplified fragments are indicated for each Line_x case. Δ, big deletions / insertions. Cx denotes the colony number of the sequenced bacterial clone resulting from the PCR band cloning process.

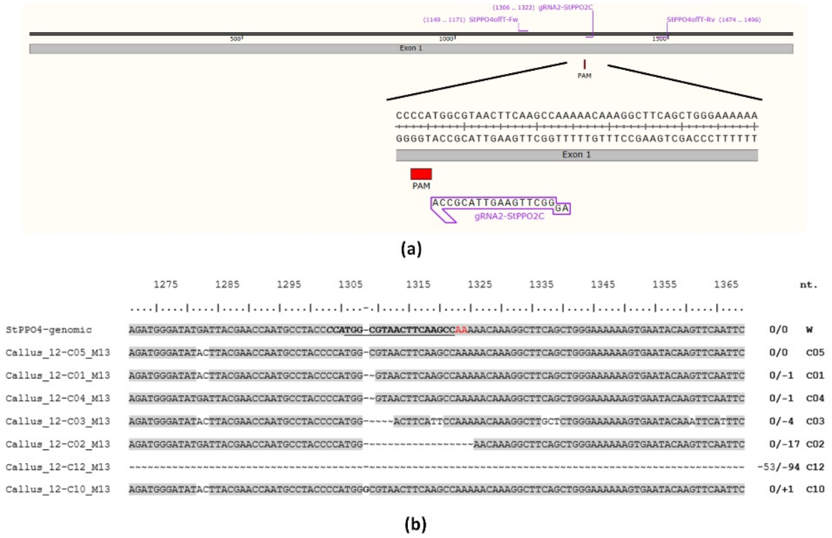

Figure S7. Off-target prediction for the used gRNAs. From the selected gRNAs in this work, only gRNA2-StPPO2C was considered as molecule with a potential off-target site with inclusion of three or less potential mismatches at a predicted zone in the *StPPO4* gene (**a**) which was experimentally established (**b**).

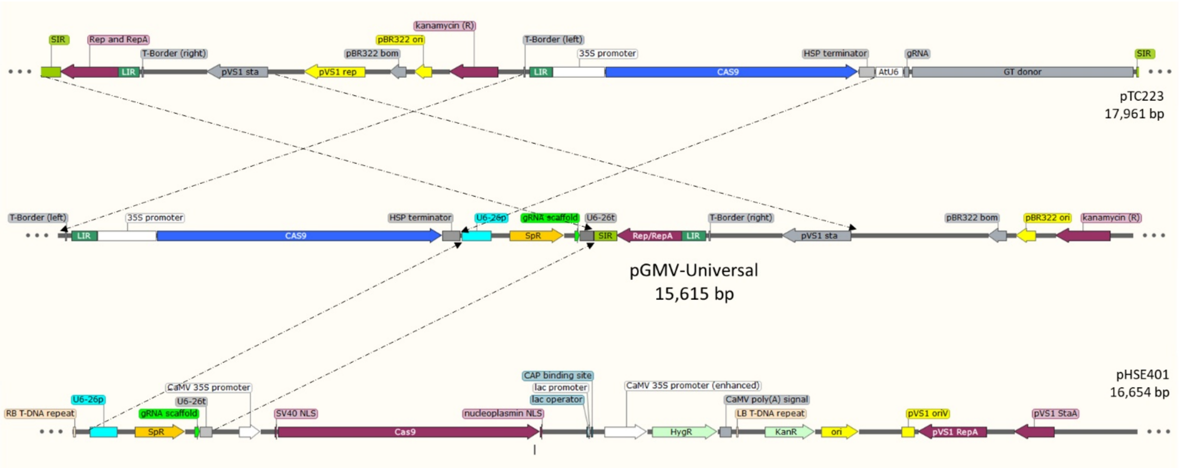

Figure S8. Universal geminivirus-based vector (pGMV-U) for the expression of multiple guide RNAs. The fragment carrying the sequence required for the insertion of multiple gRNAs was amplified from the pHSE401 plasmid, while the three remaining fragments containing sequences for the LSL replicon located within an *Agrobacterium* T-DNA sequence and the binary vector backbone were amplified from the pTC223 plasmid. Map: T-DNA RB, right border of the *Agrobacterium* T-DNA; LIR, large intergenic region from the *Bean yellow dwarf virus* (BeYDV); CaMV 35S, 35S promoter from *Cauliflower mosaic virus*; Cas9, Cas9 *Arabidopsis thaliana* codon usage; HSP terminator, terminator for *HEAT SHOCK PROTEIN 18.2* gene from *A. thaliana*; U6-26p, *A. thaliana* U6-26 RNA polIII promoter; SpR, spectinomycin resistance gene; Kanamycin (R), kanamycin resistance gene; gRNA scaffold, gRNA scaffold sequence; U6-26t, *A. thaliana* U6-26 RNA polIII terminator; SIR, short intergenic region from BeYDV; Rep/RepA, nucleotide sequence for the Rep/RepA replication genes; LB T-DNA, left border of the *Agrobacterium* T-DNA. Dotted lines represent Gibson Assembly [48] cloning from each source vector.

Table S1. GFP transient expression in 'Yagana-INIA' explants 7 days post-infection with pGEF-U.

**Explant type**	**Initial Explants**	**GFP+ (%)**	**GFP+ in PSM400cc (%)**
**Leaves**	96	57 (59.4)	26 (27.1)
	155	75 (48.4)	20 (12.9)
	108	77 (71.3)	59 (54.6)
**Internodes**	145	6 (4.1)	0 (0)
	101	1 (1.0)	0 (0)
	73	7 (9.6)	0 (0)

Table S2. Gene codes.

**Gene Name**	**Gene Code**	**Chromosome**
StvacINV1	PGSC0003DMT400035987	ch03
StBAM1	PGSC0003DMG400001549	ch09
StPPO1	PGSC003DMT400076054	ch08
StPPO2	PGSC0003DMT400048684	ch08

Table S3. Identity comparison of the targeted regions between 'Yagana-INIA' and Double Monoploid (DM) reference genome.

Region targeted by:	Check Edition (CE) primer pair used for amplification of gRNA surroundings	Amplicon Identity Yagana/DM	gRNA Identity in Yagana
**gRNA1-StvacINV1B**	StvacINV1-CEb-Fw/ StvacINV1-CE-Rv4	99,8%	100%
**gRNA2-StvacINV1B**	StvacINV1-CE-Fw3/ StvacINV1-CEb-Rv	99,7%	100%
**gRNA1-StBAM1A**	StBAM1-CEb-Fw/ StBAM1-CE-Rv3	98.9%	100%
**gRNA2-StBAM1A**	StBAM1-CE-Fw3/ StBAM1-CEa-Rv	99%	100%
**gRNA1-StPPO1B**	StPPO1-CEa-Fw/ StPPO1-CEb-Rv	98,5%	100%
**gRNA2-StPPO1C**	StPPO1-CEa-Fw/ StPPO1-CEb-Rv	98,5%	100%
**gRNA1-StPPO2C**	StPPO2-CEa-Fw/ StPPO2-CEa-Rv	97,1%	100%
**gRNA2-StPPO2C**	StPPO2-CEa-Fw/ StPPO2-CEa-Rv	97,1%	100%

Table S4. Primer Sequences.

Primer Name	Primer Sequence 5′->3′
**GWcst-Fw**	attaggcgcgccGTCTCAGAAGACCAAAGGGC
**GWcst-Rv**	TCGGGAATTAATAGAAATAGGCG
**AscI-Upstream**	CGACCCTCGGTACCGATCGG
**AscI-Downstream**	CTGAAATTGAAAAGGCAAATCTGGCGCG
**gmv-CI-Fw**	TGAAGTACACTCGGTCAAGCT
**gmv-CI-Rv**	TCAAAAGTCCCACATCGCTTAGA
**M13-Univ-Fw**	CACGACGTTGTAAAACGAC
**M13-Univ-Rv**	CAGGAAACAGCTATGAC
**attB1-CaMV35Sx2**	ggggacaagtttgtacaaaaaagcaggcttcCCCCAGATTAGCCTTTTCAATTT
**attB2-CaMV35Sx2**	ggggaccactttgtacaagaaagctgggtcCCCGTGTTCTCTCCAAATGAAATG
**StvacINV1-CEb-Fw**	AAAACTCCGCCTCCCATTAC
**StvacINV1-CE-Rv4**	TTGAGGTTGAAAATGGTAAGCA
**StvacINV1-CE-Fw3**	CTTTTGCCATGGTTCCTGAT
**StvacINV1-CEb-Rv**	CACTCCCACATACCCGTACC
**StBAM1-CEb-Fw**	GCAATGAGTCTGCCACACC
**StBAM1-CE-Rv3**	TTTTCGCCATTTCCATAAGC
**StBAM1-CE-Fw3**	TAGCTTACAGGGTGCAGCAG
**StBAM1-CEa-Rv**	ATGCCACTTGCCTAACCAAC
**StPPO1-CEa-Fw**	GCACCATTAGCCTCTGCTTC
**StPPO1-CEb-Rv**	GCATGGGAGCGTAACTGAAT
**StPPO2-CEa-Fw**	GTGGTGCCGTACAGTTGTTG
**StPPO2-CEa-Rv**	CCACGTTCAAAAACACATCG
**StvacINV1-CEa-Fw**	CCGATCCTCAACAACCAATC
**StvacINV1-CEa-Rv**	AACCCAATTCCACAATCCAA
**StPPO4offT-Fw**	AGCAATTGGAGGTAAACGAACAG
**StPPO4offT-Rv**	TCATTGAACGTTAGCATCTCCTC
**eGFP-nst1-fw**	CACATGAAGCAGCACGACTT
**eGFP-nst1-rv**	AGTTCACCTTGATGCCGTTC
**pGEF-recircle-fw**	TGAGCACTTGGGATAGGTAAG
**pGEF-recircle-rv**	GCGCGCGGTGTCATCTATG

Table S5. Primers for gRNA1-gRNA2 module amplification.

gRNA-Primer Name	Sequence 5′->3′
**DT1-BsF-StvacINV1B**	ATATATGGTCTCGATTGTAGTCACGTTTCTTATGCGGTT
**DT1-F0-StvacINV1B**	TGTAGTCACGTTTCTTATGCGGTTTTAGAGCTAGAAATAGC
**DT2-R0-StvacINV1B**	AACATCTTAGACCCGATTGTTACAATCTCTTAGTCGACTCTAC
**DT2-BsR-StvacINV1B**	ATTATTGGTCTCGAAACATCTTAGACCCGATTGTTACAA
**DT1-BsF-StBAM1A**	ATATATGGTCTCGATTGTGAGTCTGCCACACCAGATGTT
**DT1-F0-StBAM1A**	TGTGAGTCTGCCACACCAGATGTTTTAGAGCTAGAAATAGC
**DT2-R0-StBAM1A**	AACTGGCGGGCAAGCATCTGGGCAATCTCTTAGTCGACTCTAC
**DT2-BsR-StBAM1A**	ATTATTGGTCTCGAAACTGGCGGGCAAGCATCTGGGCAA

**DT1-BsF-StPPO1B**	ATATATGGTCTCGATTGTTGGCTACGAGTCGAATGAGTT
**DT1-F0-StPPO1B**	TGTTGGCTACGAGTCGAATGAGTTTTAGAGCTAGAAATAGC
**DT2-R0-StPPO1C**	AACTCCCAATTCTTCGGTGCTGCCAATCTCTTAGTCGACTCTAC
**DT2-BsR-StPPO1C**	ATTATTGGTCTCGAAACTCCCAATTCTTCGGTGCTGCCAA
**DT1-BsF-StPPO2C**	ATATATGGTCTCGATTGTCACGTGTTACATCGAAAAGTT
**DT1-F0-StPPO2C**	TGTCACGTGTTACATCGAAAAGTTTTAGAGCTAGAAATAGC
**DT2-R0-StPPO2C**	AACTGGCGTAACTTCAAGCCCTCAATCTCTTAGTCGACTCTAC
**DT2-BsR-StPPO2C**	ATTATTGGTCTCGAAACTGGCGTAACTTCAAGCCCTCAA

Table S6 Comparison between wild type and edited amplicon size.

Gene	Check Edition (CE) primer pair	Wild type Amplicon Size (bp)	Predicted Edited Amplicon Size (bp)
StvacINV1	StvacINV1-CEb-Fw/ StvacINV1-CEb-Rv	2359	362
StBAM1	StBAM1-CEb-Fw/ StBAM1-CEa-Rv	2277	121
StPPO1	StPPO1-CEa-Fw/ StPPO1-CEb-Rv	1027	593
StPPO2	StPPO2-CEa-Fw/ StPPO2-CEa-Rv	1222	680

Table S7. Off-target analysis.

gRNA	Off-target pattern*	Off-target sequence (with 3 or less mismatches)	Off-target location (with 3 or less mismatches)
**gRNA1-StvacINV1B**	0,0,0,**1**,5	TAGGCAGGTTTCTTTTGCG	Intergenic Ch05: 15670727
**gRNA2-StvacINV1B**	0,0,0,**0**,**10**		
**gRNA1-StBAM1A**	0,0,0,**0**,8		
**gRNA2-StBAM1A**	0,0,0,**1**,6	CTCAGATGCTTACCCGCCA	Intergenic Ch07: 48748698
**gRNA1-StPPO1B**	0,0,0,**1**,9	TTGGCCATGAGTCGACTGA	Intron Ch01: 12810366
**gRNA2-StPPO1C**	0,0,0,**0**,8		
**gRNA1-StPPO2C**	0,0,0,**0**,**13**		
**gRNA2-StPPO2C**	0,0,0,**1**,**14**	TTGGCTTGAAGTTACGCCA	PGSC0003DMG400018917 Ch08: 45869470

*(a,b,c,d,e) key according to description in Materials and Methods.

Table S8. Media composition.

Medium	Composition
**Potato Propagation Medium (PPM)**	MS 2.2 g/L + Sucrose 20 g/L + Agar 7 g/L Adjust to pH 5.7
**PCM Liquid Medium (PLM)**	MS 4.71 g/L + Sucrose 20 g/L + 2,4-D 2 mg/L Adjust to pH 5.8 ***+ Zeatin 0.5 mg/L***
**High Hormone Preculture Medium (HH)**	MS 4.43 g/L + Sucrose 30 g/L + NH_4NO_3 80 mg/L + $CaCl_2$*H_2O 14.7 mg/L +NAA 10 mg/L + BAP 10 mg/L Adjust to pH 5.7
**Co-culture Potato Callusing Medium (Co-PCM)**	MS 4.71g/L + Sucrose 20 g/L + MES 500 mg/L + 2,4-D 2 mg/L + Agargel 5 g/L Adjust to pH 5.7 ***+ Zeatin 0.5 mg/L***
**Potato Callusing Medium (PCM400cc)**	MS 4.71 g/L + Sucrose 20 g/L + MES 500 mg/L + 2,4-D** 2 mg/L + Agargel 5 g/L Adjust to pH 5.7

	+ Zeatin 0.5 mg/L + Carbenicillin 400 mg/L + Cefotaxime 400 mg/L
**Potato<br>Shooting Medium (PSM400cc)**	MS 4.71 g/L + Sucrose 20 g/L + Agargel 5 g/liter<br>Adjust to pH 5.7<br>***+ GA_3 2 mg/L+ Zeatin 0.5 mg/L***<br>***+ Carbenicillin 400 mg/L + Cefotaxime 400 mg/L***
**Potato<br>Rooting Medium<br>(PRM100c)**	MS* 4.71g/L + Sucrose 20 g/L<br>+ Agargel 5 g/L +<br>Adjust to pH 5.7<br>+ ***Cefotaxime 100 mg/L***
**LB Solid**	Tryptone 10 g/L + NaCl 10 g/L + Yeast extract 5g/L + Agar 15 g/L
**LB Liquid**	Tryptone 10 g/L + NaCl 10 g/L + Yeast extract 5 g/L

MS: Murashige and Skoog with vitamins; 2,4D: 2,4-Dichlorophenoxyacetic acid; NAA: 1-Naphthaleneacetic acid; BAP: 6-Benzylaminopurine; GA_3: Giberellic Acid; ***"added after autoclaving".***
